# Room-temperature soft mode and ferroelectric like polarization in SrTiO_3_ ultrathin films: Infrared and *ab initio* study

**DOI:** 10.1038/s41598-017-02113-4

**Published:** 2017-05-19

**Authors:** Wei-wei Peng, Robert Tétot, Gang Niu, Emilie Amzallag, Bertrand Vilquin, Jean-Blaise Brubach, Pascale Roy

**Affiliations:** 1 Synchrotron SOLEIL, L’Orme des Merisiers, Saint-Aubin, BP 48, F-91192 Gif-sur-Yvette, France; 2 CNRS-Université paris-Sud, ICMMO(SP2M) UMR 8182, Bât 410, F-91405 Orsay Cedex, France; 3 Electronic Materials Research Laboratory, Key Laboratory of the Ministry of Education & International Center for Dielectric Research, Xi’an Jiaotong University, Xi’an, 710049 China; 4 Ecole Centrale de Lyon, Institut des Nanotechnologies de Lyon (INL), Université de Lyon, CNRS-UMR 5270, 36 Avenue Guy de Collongue, F-69134 Ecully, France

## Abstract

Due to the remarkable possibilities of epitaxially growing strontium titanate (SrTiO_3_ or STO) on silicon, this oxide is widely used as a buffer layer for integrating other perovskite oxides which allows for the development of various functional electronic devices on silicon. Moreover, STO is known to be an incipient ferroelectric in bulk but may become ferroelectric when in the form of strained ultrathin films. Given the importance of the potential applications for electronics if this property is demonstrated, we performed a spectroscopic study of STO on Si(001) templates coupling experimental and ab initio investigations. We selected six samples of ultrathin films: three strained samples (of thickness 4, 9 and 48 nm) and three relaxed samples (of equivalent thickness). Their infrared spectra show that both the mechanical stress and the thickness play major roles: higher energy modes evolve as soft modes in thinner strained films. In order to support these observations, the dynamical *ab initio* calculations allowed deriving the conditions for STO films to become ferroelectric at room temperature as shown by the development of a soft mode and the divergence of the in-plane dielectric constant.

## Introduction

Perovskite oxides form a class of electronic materials which covers a wide range of electrical properties such as superconductivity, piezoelectricity, ferroelectricity and ferromagnetism. Various functional devices, like electromechanic captors, electro-optic couplers, ferroelectric non-volatile memories, radio-frequency filters etc., based on perovskite oxide thin films grown on SrTiO_3_/Silicon (STO/Si) templates are developed around these properties^1, 2^. Substrate-like STO/Si(001) has been the subject of several studies motivated by its potential application as a template to integrate functional oxides with perovskite structure on silicon^3–6^. Indeed, very recent works have shown that STO/Si crystalline templates could be used for the monolithic integration of III–V semiconductors on silicon, due to the properties of the semiconductor/STO heterointerfaces^7–9^.

Concurrently, STO itself is very attractive for technological and fundamental research as a system combining structural and ferroelectric instabilities^10^. STO undergoes a cubic to tetragonal (I4/mcm-D_4h_) phase transition at around 105 K, with a small tetragonal distortion (c/a = 1.0015) at 10 K^11^, connected with an antiferrodistorsive soft phonon mode. STO also undergoes a pressure-induced cubic to tetragonal transition at 10 GPa at room temperature^12^. On the other hand, STO bulk presents a remarkably high static dielectric constant at room temperature (~300 K) which increases by about a factor of 10^2^ at 4 K. However, the phonon mode responsible for this ferroelectric (FE) behavior does not condensate at any temperature due to quantum fluctuations, which makes STO a so-called incipient ferroelectric, with a critical temperature T_c_~32 K^13–15^. Nevertheless, it has been shown that the FE phase can be induced in bulk STO by chemical pressure (^18^O substitution^16, 17^, Ca doping^18^) or surface effects^19^. Unfortunately, in STO thin films, the dielectric constant tends to be significantly reduced, thereby limiting their applications. This size effect is not fully understood and may be due, according to the authors, to a dead layer effect^20, 21^, internal stress^22^ or/and a profound change of the lattice dynamical properties resulting in the hardening of the soft mode^23–26^. In contrast, many theoretical and experimental studies have shown that it is possible to produce FE in STO ultra-thin films by a misfit-strain even at room temperature^27–32^. Notice however that, the values of critical strain and temperature reported in these studies are not consistent amongst each other and that the film thickness which can significantly affect the mechanical stress of the film is not always specified in these studies.

Obviously, determining the conditions for obtaining ferroelectric STO thin films directly on silicon at room temperature is crucial for technological applications. Very interestingly, Warusawithana *et al*.^30^ observed, by means of piezoresponse force microscopy (PFM), FE nanodomains at temperature as high as 400 K in STO ultrathin films (2 to 4 nm) grown on Si (001) substrates, while such domains were no longer observed for thicker 8 nm films. PFM is widely used to study ferroelectricity in thin films as it allows to write, read and reversibly switch polar domains. Such domain structures can be observed using conventional scanning probe techniques of optical second harmonic generation (SHG)^30, 32–34^. Alternatively, in this paper, we investigate the ferroelectricity in strained STO by means of the spectroscopic study of the soft mode. The soft mode concept was proposed by Cochran^35^ in 1960 and it has since been used as a non-ambiguous signature of FE transitions^13, 23–26^. In order to define the specific roles of strain and thickness for developing FE properties at room temperature in STO/Si(001) ultrathin films, we report here (i) the first observations of the phonons and soft modes on such nanometer thick samples where ferroelectric transition is expected^36^ and (ii) DFT calculations of the dielectric constant and phonon modes for 1.2, 2.0, 2.5 nm thin films for various strain values. Absorbance measurements have been carried out between 20–600 cm^−1^ on six STO/Si(001) ultrathin films (two series of three films ∼4, 10 and 50 nm). One series underwent an annealing after deposition to partially relax the strain induced by the −1.69% mismatch between the cubic STO parameter (a[100] = 3.905 Å) and the Si(001) parameter (a[110] = 3.840 Å). The other series was as-grown on Si and may retain some strain induced by the STO/Si(001) mismatch. The strain of the as-grown and unstrained samples were determined by RHEED, XRD (in plane and out of plane) and TEM. In short, the in-plane strain of the as-grown films of 4 nm, 10 nm and 50 nm can be estimated as −1.69%, −1.4% and 0% respectively while all unstrained samples present no detectable in-plane strain.

The synthesis of films, strain determination and details of IR spectra measurements and DFT calculations are reported in Methods.

## Experimental and calculated IR spectra

**Figure 1:**
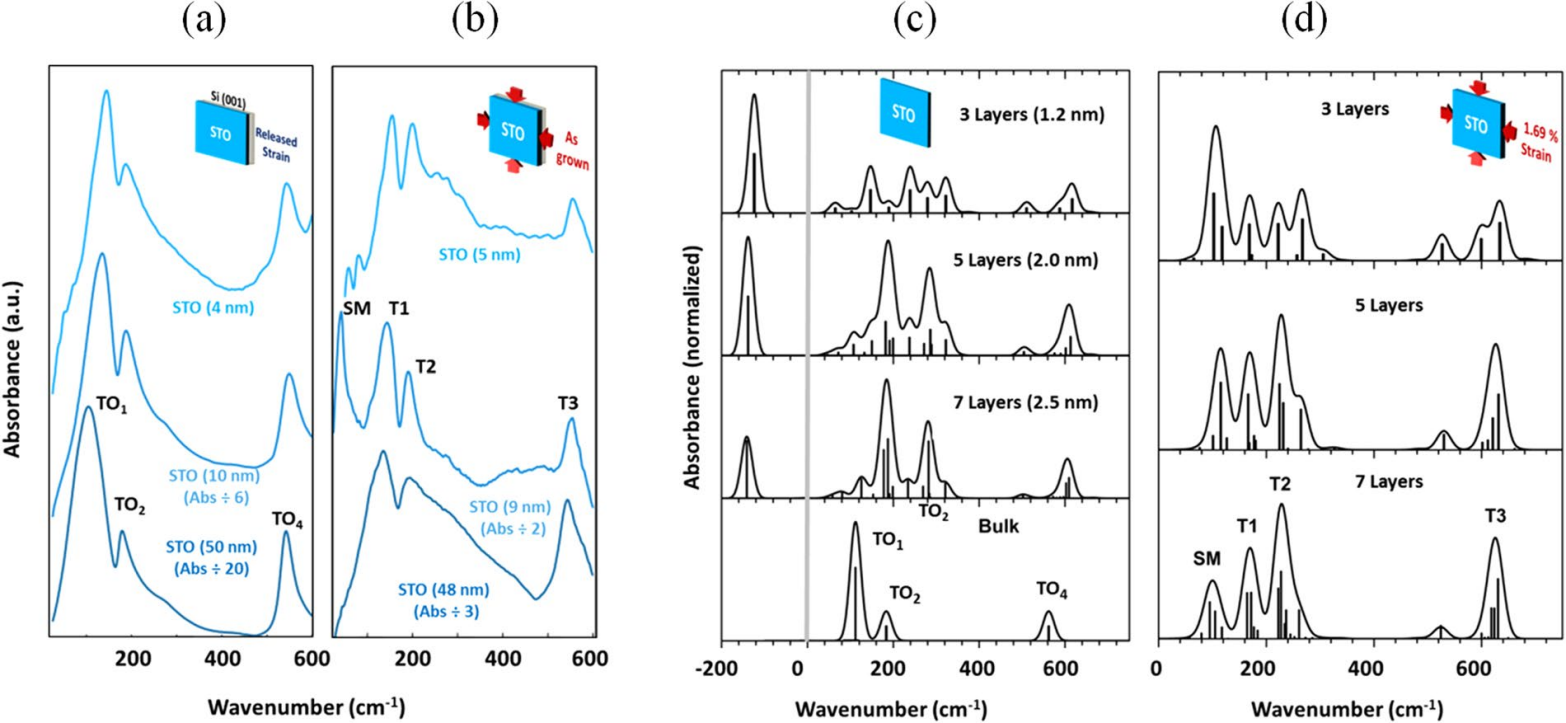
Measured and calculated absorbance of STO thin films with various thicknesses and strain states. (**a)** IR absorbance of the unstrained STO/Si(001) films of thickness 50, 10 and 4 nm. The three phonon structures TO_1_, TO_2_ and TO_4_ correspond to the classification of the bulk phonon modes. (**b)** IR absorbance of the strained (as-grown on Si) STO/Si(001) films of thickness 48, 9 and 5 nm. The four main structures of the intermediate film are labelled SM, T1, T2, T3, where SM designates surface and/or soft mode and T1, T2, T3 bulk-like modes (see details in the text). Differences in the spectra of the two thicker films may be related to the difference in their relaxation mechanism (See refs 40 and 41). The plastic relaxation of the as-grown 48 nm film causes small domains bordered by dislocations at the interface. (**c**) Calculated absorbance of STO bulk and fully relaxed STO slabs of 7 layers (2.5 nm), 5 layers (2 nm) and 3 layers (1.2 nm). The presence of modes at negative values (imaginary modes) demonstrates that all slabs are metastable. (**d**) Calculated absorbance of STO slabs of 7, 5 and 3 layers with −1.69% strain corresponding to the lattice mismatch between STO and Si(001). Modes are labelled as in (**b)**. In (**c**–**d)** sticks are proportional to oscillator strength (maximum intensity normalized at 1) and lines represent the sum of 30 cm^−1^ wide Gaussians each centered at the modes frequencies.

The measured IR spectrum of the three unstrained and of the three strained STO/Si(001) films are presented in Fig. 1a and b, respectively. It can be observed first that the experimental spectra appear to be strongly strain dependent, especially for the two thinner layers (∼10 and 5 nm). All six films show three main phonon modes. The unstrained and strained thickest films (∼50 nm) present the so-called TO_1_, TO_2_ and TO_4_ peaks as observed for bulk^13–15, 37^ (See Table 1). For bulk modes, the assignments can be resumed as follows: TO_4_ and TO_2_ are Ti-O stretching and bending modes ﻿respectively, while the Sr-TiO_3_ lattice vibration gives rise to TO_1_^37^. Meanwhile, two weaker bands appear on the thinnest strained film at 58 and 81 cm^−1^ and an intense band is clearly present on the strained 9 nm film at ~40 cm^−1^. In short, the strained thinner layers (5 and 9 nm) present phonons structures shifted at higher frequency and extra phonon at low frequency (probably due to surface modes) compared to bulk or to thicker relaxed films. Figure 1c presents the calculated phonon structures for bulk (bottom), and unstrained slabs of 7, 5 and 3 STO layers (∼2.5, 2.0 and 1.2 nm respectively). Note that the 7 layers film presents a thickness (2.5 nm) comparable to our thinnest measured films (4 nm). This thickness was chosen as it allowed a reasonable duration of the calculations. As a matter of fact, the loss of symmetry in the direction perpendicular to the slab makes the calculations of IR spectra extremely CPU time consuming. The simulated spectra show an excellent agreement with measurements for bulk^13–15, 37^ as well as for the thicker films (See Fig. 1a and b and Table 1). Certainly, in terms of phonon positions and relative intensities both experimental and simulated spectra include three phonon structures, two weaker around 200 cm^−1^ and 550–600 cm^−1^ and a stronger one around 100 cm^−1^. Indeed, the numerous modes for the various slabs result from the appearance of surface modes. It is worth highlighting that the calculated spectra of free slabs exhibit an intense imaginary mode (∼140i), showing that unstrained slabs are metastable. Figure 1d presents equivalent calculations on slabs of the same thickness under −1.69% strain. Notice that imaginary modes of Fig. 1c are no longer present in the calculated spectra showing that strain has stabilized these slabs. Compared to relaxed slabs, the main spectral features appear qualitatively in the same energy range but intense phonons are present at low frequencies. Most peaks, under 400 cm^−1^, result from the lowering of the symmetry of slabs compared to bulk which generates multiple components from TO_1_ and TO_2_ modes. This larger number of modes is in good agreement with the rich experimental 5 and 9 nm strained films spectra although none of the simulated spectra presents mode under 50 cm^−1^.

**Table 1:** Measured and Calculated Frequencies of the main modes for ultrathin films and Bulk together with some assignment.

Vibrational modes	Measured Frequency (cm^−1^)							Calculated Frequency (cm^−1^)			**Main Assignment (from calculations)**		
	Film 4 nm		Film 10 nm		Film 50 nm		Bulk (Petzelt^14^)	Bulk	7 slabs		**Bulk**	**7 slabs**	
	relaxed	strained	relaxed	strained	relaxed	strained			relaxed	strained (−1305%)		**relaxed**	**strained (−1.305%)**
TiO_2 Surface_ Transverse/Longitudinal modes													
SM	50	58		42				…		28	**…**		(S) Ti-O
	65	81						…		45	**…**		(S) Ti-O
								…	**141i**	62	**…**	(S) Ti-O	(B) O-Ti-O
SrO Surface Transverse/Longitudinal modes													
SM									80	164/	**…**	(B) Sr-O-Sr	(B) Sr-O-Sr
									234	260/	**…**	(B) O-Ti-O	(B) O-Ti-O
Transverse/Longitudinal modes (Bulk like)													
T 1	143	155	133	143	133	105	93	112/846	185	171	(S) Sr-OTi (B) O-Ti-O	(B) O-Sr-O	(B) O-Sr-O
T 2	187	203	189	191	189	179	176	183/171	282	**230/5700**	(B) Ti-O-Ti	(B) Sr-O-Sr	(B) O-Ti-O
T 3	544	555	547	553	547	542	548	562/489	609	620	(S) Ti-O (B) Sr-O-Ti	(B) Ti-Sr-Ti (S) Ti-O	

Left: Measured frequencies of the main optical modes for strained and relaxed STO thin films of various thicknesses (~50, 10 and 4 nm) together with experimental bulk frequencies from the literature^37^.

Center: Calculated frequencies of the main optical modes for −1.305% strained and relaxed STO slab of 7 layers together with calculated bulk frequencies. All modes are divided into Transverse Modes T1, T2 and T3 and Surface/Soft Modes (SM). SM are classified according to STO terminated surface: SrO or TiO_2_.

The presence of imaginary mode at 141i (in bold) shows that the relaxed slab is metastable. The modes contributing to the large dielectric constant for strained slabs (surface modes and the large LO/TO splitting T2 mode) also appear in bold.

Right: The assignment based on calculations of the main optical modes for strained and relaxed together with calculated bulk frequencies are presented in the three last columns.

### Calculated spectra and dielectric constant of STO ultrathin films according to the strain

**Figure 2:**
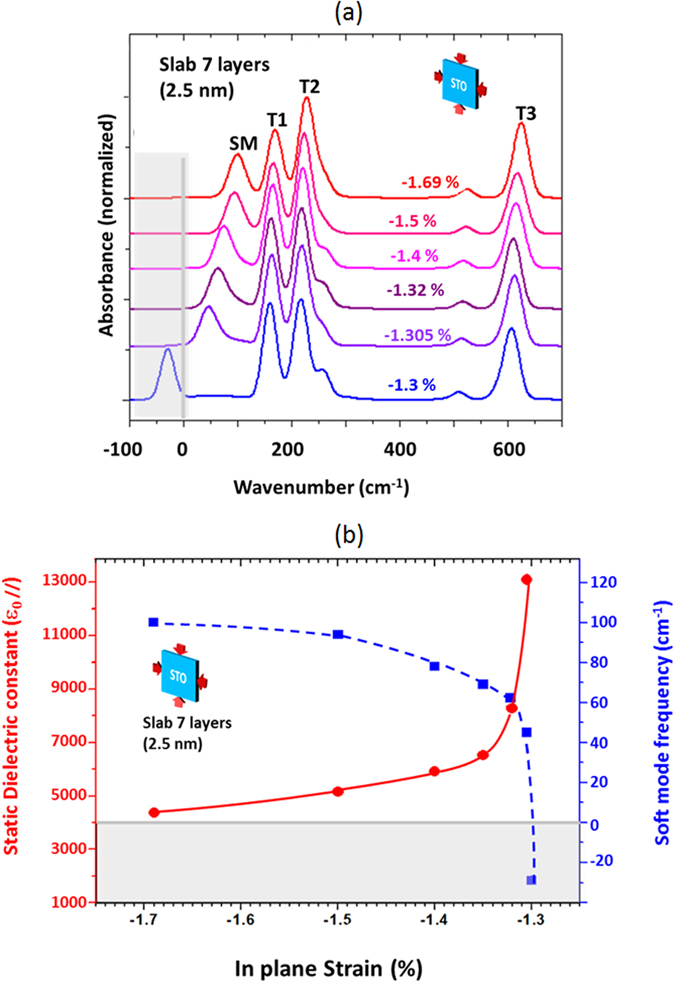
Results of DFT calculations for a STO slab of 7 layers under various in-plane strains between −1.69% and −1.30%. (**a**) Simulated spectra for various strains. A very strong dependence of the Surface/Soft mode (SM) passing from ~100 cm^−1^ to an imaginary value as the strain decreases is observed. At −1.305% strain, the SM is located at 40 cm^−1^ while it becomes imaginary for strain of −1.3%. (**b**) Variations of the Soft Mode frequency and of the static dielectric constant parallel to the slab as a function of the strain. The values of both the soft mode and the in-plane static dielectric constant diverges for strain values under −1.305%.

In the search for the conditions for developing a soft mode (and the FE state), we underwent systematic calculations on STO slab for various strain levels under −1.69% (where no imaginary mode is observed) down to the occurrence of an imaginary mode. The results are presented in Fig. 2a for the slab of 7 layers (results for 3 and 5 layers show the same trend and are not shown here). This 7 layers slab corresponds to a thickness of 2.5 nm, a value of the same order as the 4 nm samples measured here, allowing therefore a qualitative comparison between the calculated and measured spectra.

Indeed, the lowering of the parallel strain from −1.69% to −1.305% causes a progressive modification of the simulated spectra: the frequency of the SM mode tends to zero as the strain decreases down to −1.305% and becomes imaginary for −1.30%, while T1, T2 and T3 modes are independent of strain. Notice, however, that attempts to evaluate the spectra at intermediate strain failed because the structure oscillates between being stable and metastable.

Within the CRYSTAL code, the static in-plane dielectric constant $\varepsilon {(0)}_{//}$, can be evaluated for slabs under constrains as follows. First, the polarisability ${\alpha }_{//}$ (parallel to the surface) and *α*_⊥_ (normal to the surfaces) are determined as the second derivatives of the energy of the slab as a function of an external electric field (// or ⊥) according to the relationship:

$$E=E(0)-{\mu }_{i}{F}_{i}-{\alpha }_{ii}\frac{{{F}_{i}}^{2}}{2},$$

where i stands for // or ⊥ and ${\mu }_{i}=\frac{\partial E}{\partial {F}_{i}}{)}_{0}$ and ${\alpha }_{ii}=\frac{{\partial }^{2}E}{\partial {F}_{i}^{2}}{)}_{0}$. Then the tensor *ε*(∞)_*i*_ is calculated by means of the relationship$\,\varepsilon {(\infty )}_{i}=(1-\frac{4\pi {\alpha }_{ii}}{V})$, where V is the volume of the unit cell of the slab. The tensor *ε*(0)_*i*_ as well as the longitudinal optical modes (LO) are finally calculated with the CRYSTAL code using the *ε*(∞)_*i*_ tensor. The results are shown in Table 2 for bulk and strained slabs (−1.69% and −1.305%). As the strain is lowered, $\varepsilon {(0)}_{//}$ increases while *ε*(0)_⊥_ remains constant. The variations of$\,\varepsilon {(0)}_{//}$ are reported in Fig. 2b together with the soft mode frequency dependence on the strain. One can readily notice that $\varepsilon {(0)}_{//}\,$tends to infinity and the soft mode frequency tends to zero between strains of −1.305 and −1.30%, which denotes a FE state.

**Table 2:** Calculated dielectric properties of STO bulk and slab of 7 layers for −1.69% and −1.305% strains.

Bulk									
V/4π (bohr^3^)	α (bohr^3^)		ε(∞)			ε(0)		ε(0)/ε(∞)	
31.74	24.49		4.4			166		37.3	
Strained Slab of 7 layers									
Strain	V/4π (bohr^3^)	α//(bohr^3^)		α_⊥_ (bohr^3^)	ε(∞)_//_	ε(∞)_⊥_	ε(0)_//_	ε(0)_⊥_	ε(0)_//_/ε(∞)_//_
−1.69%	27.96	21.92		23.5	4.63	6.3	4672	18.1	1009
−1.31%	28.16	22.34		23.7	4.83	6.3	13267	18.1	2747

The bulk calculated values ε(∞) = 4.4 and ε(0) = 166 can be compared to the experimental value of 5.6, on the one hand, and to the experimental value of 300 and to the LST based value of 280 from ref. 15, on the other hand.

**Figure 3:**
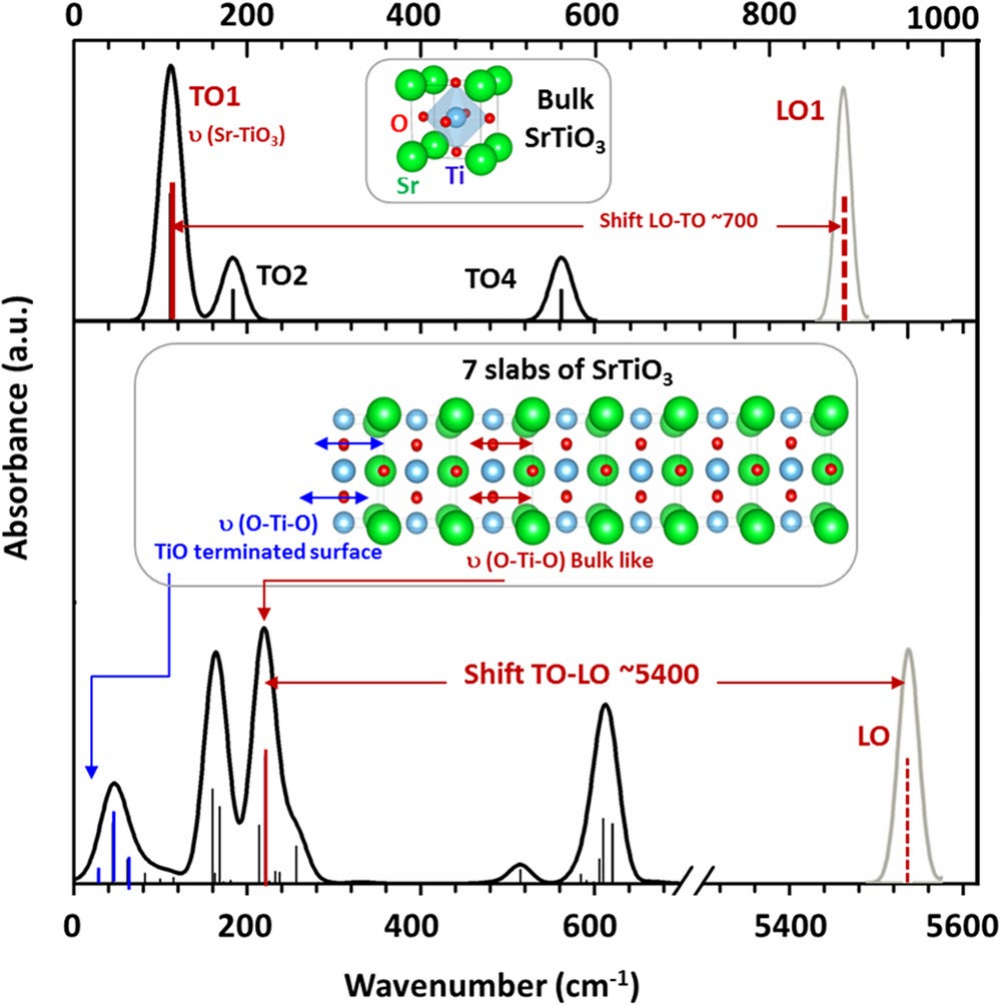
Main optical modes contributing to the large static dielectric constant for STO bulk (top) and for a slab of 7 layers under −1.305% strain (bottom). The bulk TO modes are represented as black and red sticks. Meanwhile, only the large LO-TO shift for the TO_1_ mode (shift ~700 cm) is illustrated with a red arrow. At the bottom of the figure, the main optical modes for a 7 layers slab under −1.305% strain are presented. The black sticks represent modes which derive their intensities from atoms further from the surfaces (bulk like modes). The blue sticks are transverse modes from atoms at the TiO_2_-terminated surface. The red stick at 210 cm^−1^ corresponds to a TO mode from Ti-O bending (bulk like mode). The very large LO-TO shift of the 210 cm^−1^ mode (indicated by a red arrow) contributes strongly to the high value of *ε*(0)_//_. Insets top and bottom: the elementary cell for bulk and for 7 layers slab (Sr: big green sphere, Ti: medium blue sphere, O: small red sphere).

We now focus on the origin of the divergence of $\varepsilon {(0)}_{//}$ in strained slabs. For a crystal with *N* IR-active optical modes, the Lyddane-Sachs-Teller relation (LST) connects the dielectric constant to the optical phonon frequencies:

$$\frac{{\epsilon }(0)}{\,{\epsilon }(\infty )}=\prod _{j}^{N}{(\frac{{\omega }_{L{O}_{j}}}{{\omega }_{T{O}_{j}}})}^{2}=\prod _{j}^{N}{(1+\frac{shif{t}_{j}}{{\omega }_{T{O}_{j}}})}^{2},$$

where *ω*_*LOj*_ and *ω*_*TOj*_ are longitudinal and transversal optical frequencies, respectively, and $shif{t}_{j}={\omega }_{LO{}_{j}}-\,{\omega }_{TO{}_{j}}$. This relation applied to the slab of 7 layers spectrum for −1.305% strain yields $\frac{{\epsilon }(0)}{\,{\epsilon }(\infty )}=2795$, to be compared with the value $\frac{{\epsilon }{(0)}_{//}}{\,{\epsilon }{(\infty )}_{//}}=2747$ of Table 2, which shows that the dielectric constant of the slab is mainly due to the in-plane component. From the LST relation, a very high value of *ε*(0) may arise from two effects; (i) when a particular *ω*_*TOj*_ tends to zero with a non-zero LO-TO shift, (ii) when a particular LO-TO shift tends to infinity. In the present case, both effects contribute and the very high value of $\varepsilon {(0)}_{{\parallel }}$ (13267) is mainly obtained from the contribution of four peaks: the three peaks of the surface/soft modes (SM) and the peak at 220 cm^−1^ (See Fig. 3). In Fig. 3, the main contributions to the static dielectric constant are illustrated for the bulk (top of the figure) and for the −1.305% strained slab of 7 layers (bottom of the figure). In the bulk, the lowest frequency phonon TO_1_-LO_1_ at 112 cm^−1^–846 cm^−1^ is the main contribution to the static dielectric constant. The other two (TO_2_-LO_2_ and TO_4_-LO_4_) contribute only marginally. In the strained slab, the transverse modes between 100 and 200 cm^−1^ and at ~600 cm^−1^ are caused respectively by the transformation of TO_1_ and TO_2_ (mixed contribution of Sr-TiO_3_ stretching and O-Ti-O bending for atoms located in the middle of the slab), and TO_4_ (mainly Ti-O stretching for atoms located in the middle of the slab). At lower frequencies (under 100 cm^−1^), the modes can be assigned to surface components of mixed Ti-O bending and stretching contributions parallel to the TiO_2_-terminated surface while the lowest frequency mode is a pure bending of O-Ti-O at this surface. The high value of *ε*(0)_*//*_ is due to the LO-TO shift of the 220 cm^−1^ mode (strain independent) and a smaller contribution comes from the LO-TO shifts of the three surface/soft modes (between 3 and 15), however, this contribution increases as the strain approaches −1.3% while the TO frequencies tend to zero. This second contribution is clearly at the origin of the FE transition induced by strain.

### Strain dependent Curie law for STO ultrathin films

When a ferroelectric is cooled towards the critical temperature *T*_*c*_, the soft mode frequency tends to zero, according to the Curie law:

$${\omega }_{T{O}_{SM}}(T)=const\times {\lfloor T-{T}_{c}\rfloor }^{0.5}.$$

Since the frequencies of all other phonons are almost temperature independent, the LST equation can be simplified into $\varepsilon (0)(T)\propto {\omega }_{T{O}_{SM}}^{-2}(T)$ resulting in:

$$\varepsilon (0)(T)=const\times {\lfloor T-{T}_{c}\rfloor }^{-1}.$$

**Figure 4:**
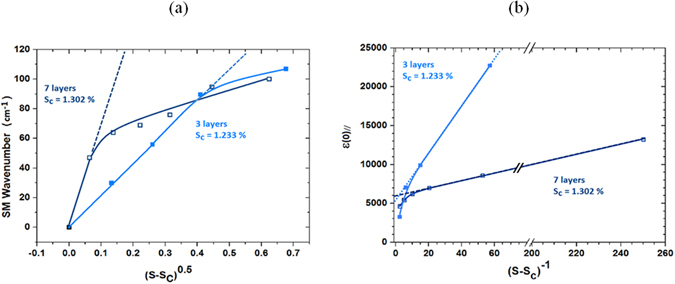
Representation of the ferroelectric Curie law as a function of strain for STO slabs of 3 and 7 layers. (**a)** Soft mode frequency dependence on the square root of strain. (**b)** Static parallel dielectric constant dependence on the inverse of strain. The dashed lines are the representations of the Curie law. One observes that the Curie law is followed in a much larger strain range for slab of 3 layers which can be explained by the much larger role of surfaces in this case.

Supposing now that the linear thermal lattice expansion *α* is a constant, the lattice parameter *a* follows a linear law: $a={a}_{0}(1+\alpha T)$ and the strain $S=\frac{a-{a}_{0}}{{a}_{0}}=\alpha T$ is proportional to *T*. Hence, the previous Curie law applies according to the strain: ${\omega }_{T{O}_{SM}}(S)=const\times {\lfloor S-{S}_{c}\rfloor }^{0.5}$ and $\varepsilon (0)(S)=const\times {\lfloor S-{S}_{c}\rfloor }^{-1}$ where *S*_*c*_ is the critical strain. This can be verified in the Fig. 4(a and b) for STO slab of 3 and 7 layers.

### Calculated polarization of the TiO_2_-terminated surface

Our calculation on −1.305% strained STO slab of 7 layers shows that the terminations present a first-layer puckering, with oxygen ions being pulled out of the surfaces by quantities s(Ti)=0.058 Å, in qualitative agreement with the experimental data of Bickel *et al*.^19^ (0.08 ± 0.08 Å). In parallel, the first two interlayers distances decrease by about 0.1 Å (*d*_0_=1.81 Å instead of 1.92 in the bulk). Following Bickel *et al*.^19^ we can evaluate the polarization of the TiO_2_ surface, *P* = *qs*/*V*, where *q*(*Ti*)~2.5 and *V* = a^2^ × *d*_0_. We obtain *P*(*Ti*) = 9.10^−6^ C/cm^2^ to be compared with the value of 24.10^−6^ C/cm^2^ for the exemplary room temperature ferroelectric BaTiO_3_, which confirms the ferroelectric character of the slab.

**Figure 5:**
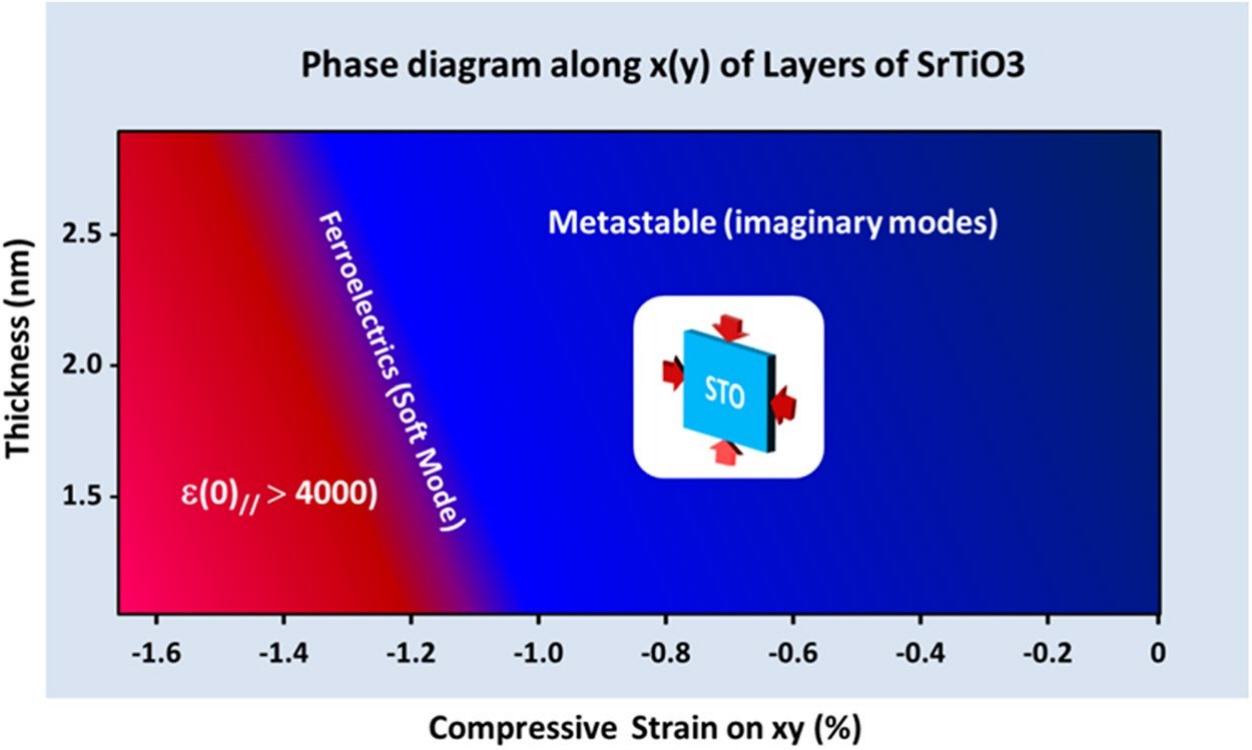
Schematic thickness-strain phase diagram based on DFT analysis for STO ultrathin films at room temperature. The strain is varied between −1.69% (Si (001) induced strain) and 0% (fully relaxed) and thickness between 0.5 and 2.5 nm. The ferroelectrics transition corresponds to the zeroing of a soft mode. The metastable phase corresponds to the development of an imaginary mode.

In conclusion, we demonstrated by means of IR measurements, DFT calculations and a soft mode analysis, the possibility of obtaining ferroelectric ultrathin film of SrTiO_3_ directly on Si(001) at room temperature. These results corroborate those by Warusawithana *et al*.^30^ obtained by means of piezoresponse force microscopy on similar samples. We found that the ferroelectricity is mainly due to the soft O-Ti-O bending mode which develops at the TiO_2_-terminated surface of the film. Two critical parameters may be controlled to obtain this result: the thickness of the film and the in-plane strain. To illustrate our results, we propose the schematic phase diagram represented in Fig. 5. The thicker the film, the larger the (compressive) strain needed to develop ferroelectricity. Concerning the thickness, IR spectra show SM modes in films of thickness 9 and 5 nm while the thicker film of 48 nm only presents higher frequency features. Such lack of SM suggests that the thicker film is somewhat too relaxed to develop FE which is in qualitative agreement with the non-ferroelectric film of 8 nm thickness reported by Warusawithana *et al*.^30^. Notice that if one extrapolates the calculated ferroelectric transition at the full compressive strain induced by the mismatch between STO and Si(001), −1.69%, the FE should develop for thickness of ~6 nm.

This study demonstrates for the first time, that a large in-plane dielectric constant can develop in ultra-thin films for strains between a critical value (~−1.23% for a thickness of 1.2 nm) up to −1.69%.

## Methods

### SrTiO_3_/Si(001) films synthesis

For this study, six STO thin films on p-type Si(001) substrates were developed by molecular beam epitaxy (MBE) as described in detail in our previous studies^38–41^. We summarize the main steps here. Sr and Ti metal effusion cells and a needle valve controlled high-purity molecular oxygen source were used. *In-situ* RHEED was employed to monitor the film surface structural properties in real time during the growth. The silicon substrate was prepared by a Sr-assisted passivation method leading to a 1/2 monolayer (ML)-covered Si surface^39^ for the subsequent STO growth. The STO films were then deposited on Si (001) at well controlled temperatures (360 and 600 °C) and under a precise control of the oxygen partial pressure. The crystalline properties of the STO film were characterized using a high resolution Rigaku four-circle diffractometer. A vacuum science workshop (VSW) XPS chamber equipped with a focused unpolarised monochromatic AlKα X-ray source (υ = 1486.6 eV) and an acceptance angle around 2° was used to study the film and the interface structure.

We determine the thickness of the STO films using High Resolution X-Ray Reflectometry (HRXRR), followed by a fast Fourier Transform (FFT) analysis from interference fringes measurements. In ref. 38, typical 2θ/ω scan around STO (002) Bragg reflection condition are reported. STO possesses a perovskite-type structure (Pm3m) and the lattice mismatch between STO (a = 3.905 Å) and Si(001) (a = 5.431/rac(2) = 3.84 Å) is 1.69% with STO unit cell rotated 45° around Si surface [001] axis. The STO (002) peak appears along with the Si(004) peak, confirming the out-of-plane epitaxial relationship STO(001)//Si(001). Furthermore, the Pendellösung fringes at the shoulder of the principal STO (002) peak, attests the good crystallinity and flatness of the STO epitaxial films. Figure 3 of ref. 38 displays the high resolution transmission electron microscopy cross-sectional view images of the 4 nm STO/Si samples grown at 360 °C. As expected^42^, the epitaxial relationship between STO and Si is [100]STO(001)//[110]Si(001) and the film presents a good structural quality and an atomically abrupt interface with silicon.

A total of six samples have been retained: three strained samples (of thickness 4 nm, 9 nm and 48 nm) obtained through a growing at 360 °C and three relaxed samples (of thickness 4 nm, 10 nm and 50 nm) grown following a two-step process. For strained samples, the temperature of 360 °C was selected as it allows for STO deposition with a good crystallization without the formation of Si oxides at the interface^38^. Concerning the two step process, two monolayers of STO were firstly deposited at 360 °C to obtain a direct STO-Si interface; then, the epitaxial temperature was increased to 600 °C allowing for a better crystallization and smaller lattice mismatch. Indeed, all SrTiO_3_/Si(001) films present good crystallization, and sharp interface. The strain details of the as-grown sample (strained sample) were obtained by RHEED, XRD (in-plane and out-of-plane) and TEM. The in-plane strain of 4, 10, 50 nm were estimated as −1.69%, −1.4% and 0% respectively as described by Niu *et al*.^41^ while all unstrained samples present no detectable in-plane strain^38, 40^.

**Figure 6:**
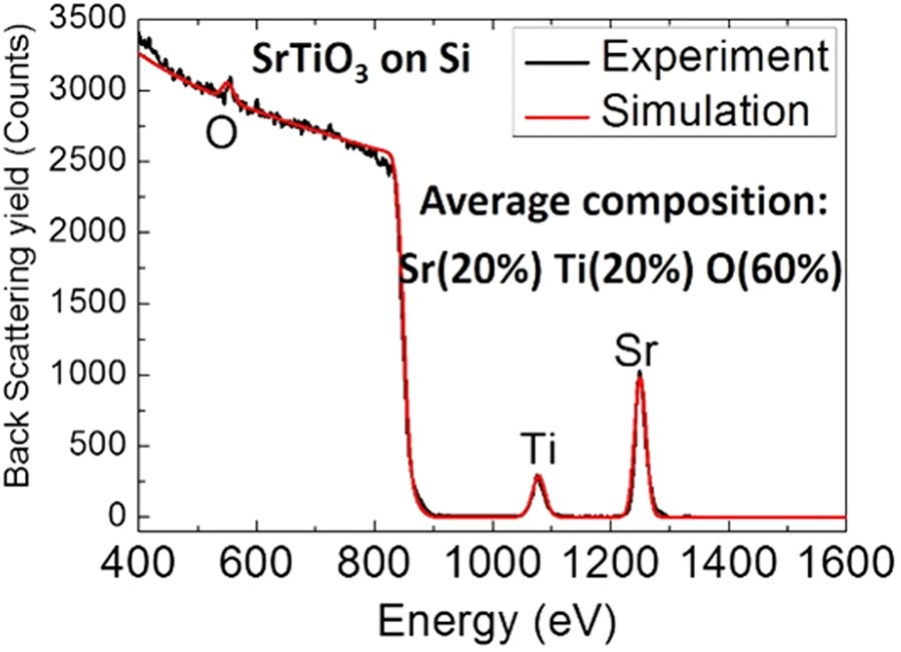
Rutherford back scattering spectra on the 10 nm thin film synthetized at 360 °C. The experiment (black curve) shows the same stoichiometry as the simulated ideal STO (red curve).

**Figure 7:**
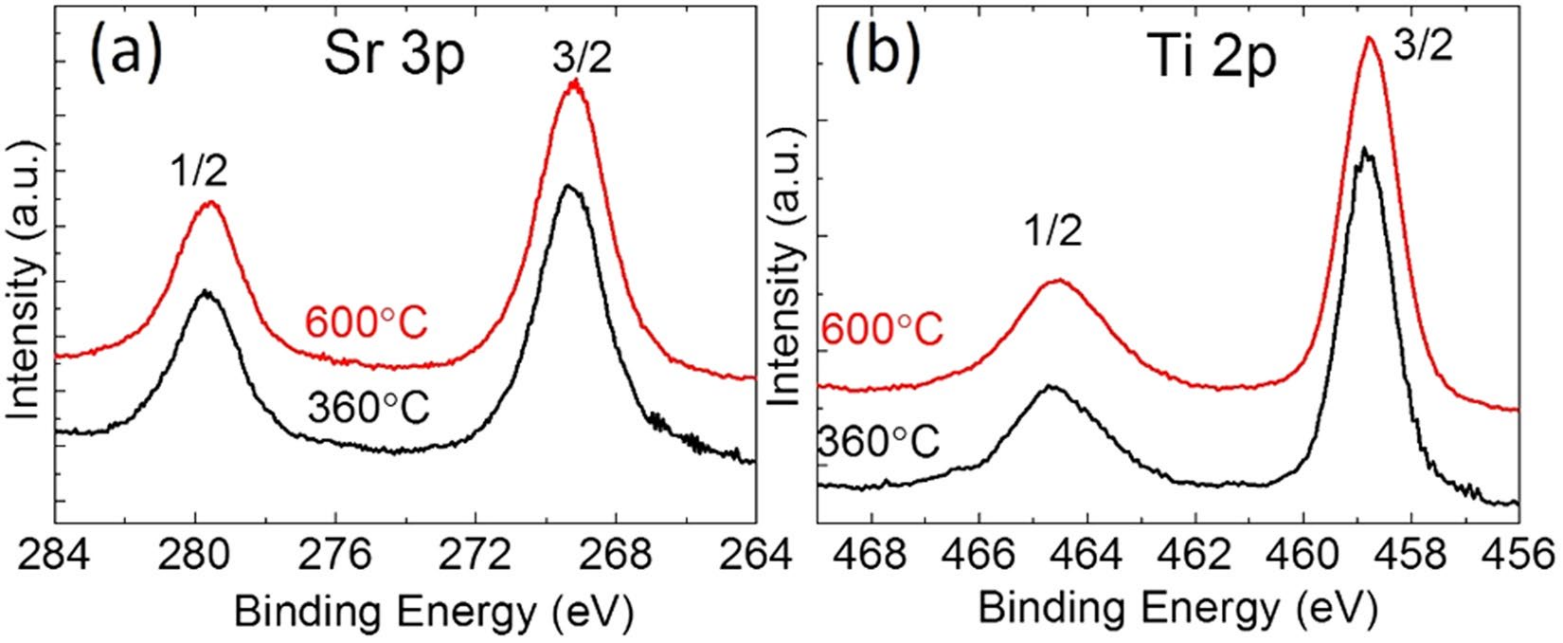
XPS spectra on 10 nm thick samples synthetized at 360 °C (black curve) and 600 °C (red curves). (**a**) zoom on the Sr3P region, (**b**) zoom on the Ti 2p region.

The cation and oxygen stoichiometry for all six samples was confirmed using Rutherford back scattering method which is bulk sensitive, and XPS which is surface sensitive. Figures 6 and 7 present examples of such measurements for the 10 nm samples. Indeed, Rutherford characterization (Fig. 1) on the 10 nm film grown at 360 °C did not detect any differences in the bulk stoichiometry compared to the ideal stoichiometry. Similarly, XPS characterization did not detect any differences in oxygen surface stoichiometry between the samples grown at 360 °C and at 600 °C. Moreover, the Ti 2p peaks in the XPS spectra (Fig. 2b) confirmed that the titanium atoms (more difficult to oxidized compared to than Sr) are fully oxidized (i.e. the sample contains a negligible amount of oxygen vacancies). Clearly, the absence of shoulder on the right side of Ti 2p 3/2 peak demonstrates the absence of oxygen vacancies.

### Infrared and THz spectroscopy

**Figure 8:**
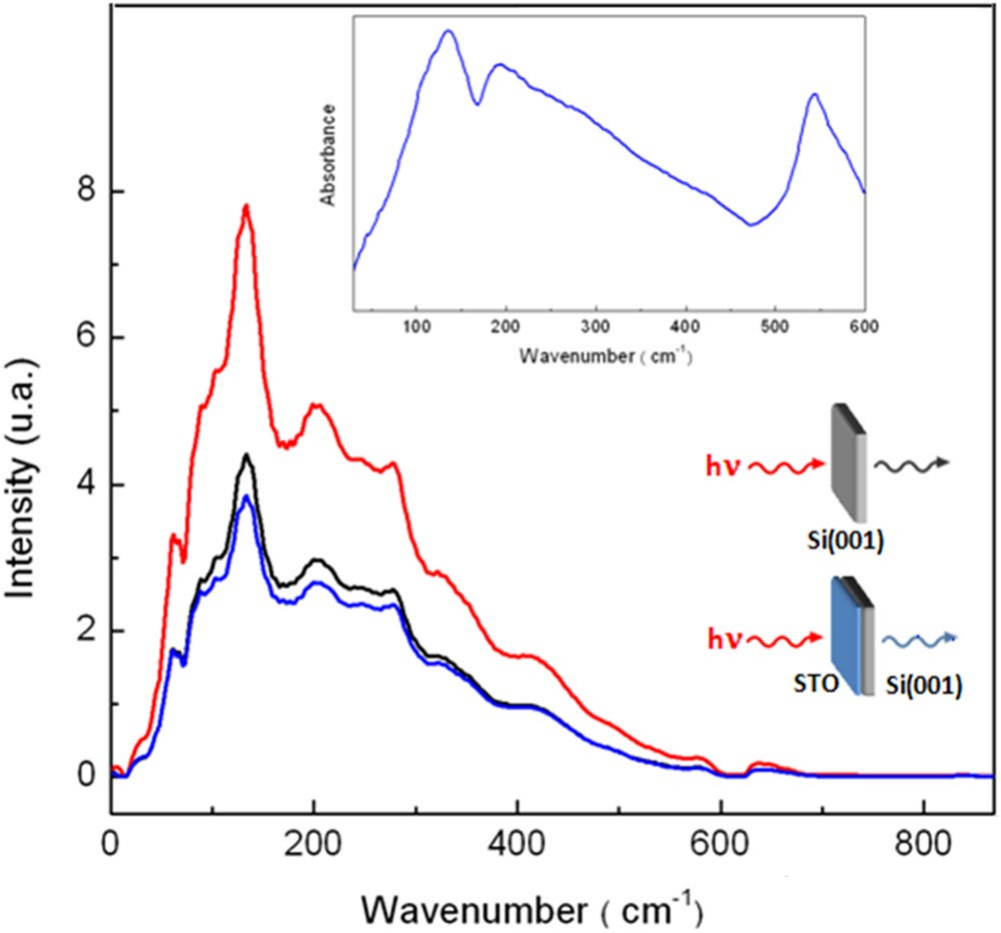
IR spectra. Incident infrared intensity (red line) and beam transmitted through a Si(001) substrate (black line) and a 50 nm STO/Si(001) film grown at 360 °C (blue line). Inset: Absorbance of 50 nm STO/Si(001) film calculated using the Si(001) substrate signal as the reference (a).

The films vibrational structures were probed using IR transmission measurements at the infrared beamline AILES (Advanced Infrared Line Exploited for Spectroscopy), Synchrotron SOLEIL^43, 44^. Exploiting the high brightness of the edge synchrotron radiation^45^, IR spectra in the 20–600 cm^−1^ range (1 cm^−1^ resolution) were obtained using a Bruker E55 Fourier transform spectrometer equipped with a 6 microns Mylar beamsplitter and combined with a bolometer (IR Lab). The interferometer and the beamline were evacuated at 10^−4^ mbar (or better) to avoid absorption by water and residual gas. The spectra of the six STO films were obtained by measuring the signal transmitted in the far infrared (FIR) and THz divided by an accurate reference provided by the signal through an equivalent Si substrate using the relation T_film_ = T_substrate+film_/T_substrate_^46^, with T the transmission. The absorbance, A, was then evaluated using the relation A = −log T. For all films measurements, a high precision sample holder allowed placing the sample and the reference at the same position relative to the incident beam which resulted in a complete compensation of the fringing from the substrate. An example of the incident beam measurement (no sample), Si(001) substrate only transmission and STO on Si(001) 50 nm film is presented in Fig. 8 together with the resulting absorbance.

### DFT calculations

The equilibrium structural parameters and the phonon modes of the IR spectra were calculated using *ab initio* calculations at the DFT level by means of the CRYSTAL09 code^47^ where the crystalline orbitals are expanded in terms of localized atomic Gaussian basis set. The B3PW functional, based on Becke’s three parameters adiabatic connection exchange functional^48^ in combination with the non-local correlation PWGGA^49^, has been used in this work.

Titanium and oxygen were treated at an all-electron level and the standard basis set HAYWSC 311(1d) G for strontium were used for orbital expansion when solving the DFT-SCF equation iteratively. The number of k points in the first irreducible Brillouin zone (Pack–Monkorst lattice)^50^ at which the Hamiltonian matrix is diagonalized is equal to 40. In optimizing the geometry, we allowed the relaxation of all atoms. A modified conjugated gradient algorithm^51^ has been implemented in the CRYSTAL code to optimize cell parameters and fractionary atomic coordinates. In geometry optimization, the criterion for convergence on the total energy is set to 10^−8^ Hartree. Γ-point vibrational frequencies and absorbance are calculated with a precision of 10 cm^−1^ on the frequency and 30% on the relative absorbance.

The full optimization of the bulk cell geometry leads to the lattice parameter a = 3.9212 Å (0.4% greater than the experimental values a = 3.905 Å) and a density of 5.06 g.cm^−3^ (1.2% greater than experimental value). The interionic distance, d_Sr-O_ = 2.773 Å and d_Ti-O_ = 1.961 Å are in good agreement (+0.4%) with experimental values (2.761 Å and 1.952 Å respectively). The Mulliken charges are respectively Q_Sr_ = 1.856, Q_Ti_ = 2.486 and Q_O_ = −1.449. The absorbance spectrum for bulk cubic STO was calculated using the relaxed structure described previously. Absorbance calculations have also been performed for slabs of 3, 5 and 7 SrTiO_3_ layers along the [001] direction (periodic boundary conditions are imposed in the [100] and [010] directions). In this case, the periodic simulation box contains a 50 nm empty space between two adjacent slabs in order to avoid surface interaction. Our results may be compared with previous theoretical studies^52–54^.
